# Outcomes of High-Dose Stereotactic Ablative Radiotherapy to All/Multiple Sites for Oligometastatic Renal Cell Cancer Patients

**DOI:** 10.3390/curroncol29100619

**Published:** 2022-10-17

**Authors:** Ming-Wei Ma, Hong-Zhen Li, Xian-Shu Gao, Ming-Zhu Liu, Huan Yin, Kai-Wei Yang, Jia-Yan Chen, Xue-Ying Ren, Dian Wang

**Affiliations:** 1 Department of Radiation Oncology, Peking University First Hospital, Beijing 100034, China; 2 Department of Health Science & Technology Strategy Information, Institute of Medical Information, Chinese Academy of Medical Sciences & Peking Union Medical College, Beijing 100006, China; 3 Department of Urology, Peking University First Hospital, Beijing 100034, China; 4 Department of Radiation Oncology, Rush University Medical Center, Chicago, IL 60612, USA

**Keywords:** stereotactic ablative radiation, stereotactic body radiation therapy, renal cell carcinoma, oligometastasis

## Abstract

Background: Stereotactic ablative body radiotherapy (SABR) is one of the treatment options for oligometastatic renal cell carcinoma (RCC) but is limited by a lack of data to evaluate high-dose SABR to all/multiple sites. Objective: This study retrospectively investigated the efficacy and prognostic factors of high-dose SABR for oligometastatic RCC patients. Design, setting, and participants: Patients with oligometastatic RCC on systemic therapy were retrospectively collected. Intervention(s): All patients were treated with SABR (40–50 Gy/5 fractions) for small tumors or partial-SABR (tumor center boosted with 6–8 Gy/3–5 fractions with 50–60 Gy/20–25 fractions to the whole tumor volume) for bulky tumors or tumors adjacent to critical organs. Outcome measurements and statistical analysis: Progression-free survival (PFS) and overall survival (OS) were calculated. Results and limitations: In total, 35 patients were enrolled, of which 88.5% had intermediate- or high-risk disease, with 60% on second- to fourth-line systemic therapy. The median follow-up time was 17 months. The median PFS and OS times were 11.3 and 29.7 months, respectively. Univariate analysis showed that an OS benefit was found in patients who received radiation before tyrosine kinase inhibitor (TKI) failure (*p* = 0.006) and where there was a short time interval (<six months) from being diagnosed with metastatic disease to undergoing radiotherapy (*p* = 0.046). Similar results were also found in PFS in patients who received radiation before TKI failure (*p* = 0.049) or within eight months (*p* = 0.047). There were certain differences in PFS (*p* = 0.033) between patients receiving radiotherapy with all lesions and those with selected tumors. In multivariate analysis, OS benefits were found in patients who received radiotherapy before TKI failure (*p* = 0.028). The limitations of this study include its retrospective design and the small patient cohort. Conclusions: The early use of high-dose SABR to multi-lesions may improve survival. Partial-SABR for bulky lesions close to critical organs could be safely and effectively applied under certain circumstances.

## 1. Introduction

Approximately 30% of renal cell cancer (RCC) patients have distant metastasis at the initial diagnosis [1]. Most study data showed progression-free survival (PFS) and overall survival (OS) of approximately one year and not exceeding three years, respectively, with targeted therapy and immunotherapy [2,3]. Intratumor heterogeneity is common in RCC patients and may explain the therapeutic failure after systemic therapy [4]. Furthermore, the large volumes of tumor lesions prevent systemic treatment from functioning to the maximum extent. In that context, an additional local approach in combination with systemic therapy is needed to eliminate metastases.

Previous studies showed that renal cancer cells are not sensitive to radiation under conventionally fractionated radiotherapy. An in vitro study showed that the capability of killing renal cancer cells was significantly enhanced with a higher single dose of radiotherapy. Furthermore, high-dose fractionation can lead to the death of tumor cells that are highly dependent on new blood vessels by damaging the tumor vascular endothelium [5], which may play a vital role in clear cell renal cell carcinoma (ccRCC) characterized by angiogenesis due to the inactivation of the von Hippel–Lindau tumor suppressor gene [6].

Stereotactic body ablative radiotherapy (SABR), which is characterized by the delivery of high doses of ionizing radiation in a few fractions, is highly effective in achieving local control, and due to the high biologically effective dose administered, it seems to overcome the radioresistance of renal cell carcinoma [7]. Although guidelines have started to recommend the use of SABR for recurrent and metastatic RCC, integrating SABR with systemic therapy as a comprehensive or upfront treatment for metastatic RCC patients is not recommended. Moreover, the appropriate dose of SABR has not yet been clearly investigated. In addition, it is not always feasible to apply SABR to bulky tumors since they may invade or be located adjacent to important normal tissues such as the intestine. To address this, we explored a new method called partial-stereotactic ablative boost radiotherapy (P-SABR). This method has been applied in the treatment of large lung tumors by our group [8]. Since the tumor volume can also be very large in mRCC, it may also be reasonable to apply this technique to metastatic renal cell carcinoma (mRCC), as well.

Here, we have introduced this novel method to mRCC patients and investigated the performance of SABR in terms of tumor control and survival in oligometastatic RCC patients. Furthermore, we have identified relevant prognostic factors that may be used to aid patient selection and the implementation of the treatment.

## 2. Material and Methods

### 2.1. Patient Population

From May 2015 to December 2020, 35 consecutive patients with oligometastases (i.e., 1–5 metastases) from histologically proven primary RCC were treated using SABR at the Department of Radiation Oncology, Peking University First Hospital. SABR was performed with a definitive intent dose to the primary tumor and oligometastatic lesions. Systemic therapy was not interrupted during or after radiotherapy in patients receiving systemic treatment.

### 2.2. Treatment Technique

High-dose SABR was performed via a gantry-mounted linear accelerator (LINAC). The internal tumor volume (ITV) was generated through 4D-CT for the primary tumor sites, lung, and liver metastases. The planning target volume (PTV) was formed with a 5 mm symmetrical expansion of the gross tumor volume (GTV) or ITV. Multiple sites were concurrently treated with SABR/p-SABR.

For primary sites or metastases, 50 Gy over five fractions were applied to the GTV or ITV and 40 Gy over five fractions to the PTV. Dose constraints for critical organs at risk (OAR) were based on the report of Task Group 101 of the AAPM [9] and the newly published article by Robert Timmerman [10]. The biological equivalent dose (BED) for a tumor ranged from 146.7 to 216.7 Gy (alpha/beta = 3).

Specifically, for large tumors (typically larger than 5 cm) or tumors close to critical organs, such as the duodenum and spinal cord, P-SABR was applied. This P-SABR plan combines a simultaneously integrated SABR boost to the maximum tumor volume with a dose of 6–8 Gy per fraction over 3–5 fractions, while the surrounding OAR dose is reduced to 3 Gy/f, followed by a conventional radiation plan consisting of at least 50–60 Gy delivered to the whole tumor volume. The detailed P-SABR plan was described in our previous study [8]. Normal tissue dose constraints for the whole P-SABR plan were based on Quantitative Analyses of Normal Tissue Effects in the Clinic [11]. The BED of the tumor center ranged from 161.3 to 185 Gy (alpha/beta = 3).

### 2.3. Endpoints

Local recurrence-free survival (LRFS), progression-free survival (PFS), and overall survival (OS) were calculated to estimate the efficacy of SABR. The time to reach the above endpoints was defined as the time from the start of radiotherapy to the time of progression or death. The independent variables considered were patients’ clinical characteristics and treatment factors. Toxicities related to SABR were assessed according to the Common Terminology Criteria for Adverse Events, v4.0.

### 2.4. Statistical Analysis

Estimates of LRFS, PFS, and OS were calculated using the Kaplan–Meier method. Hazard ratios (HRs) and associated 95% confidence intervals (CIs) were estimated with Cox’s regression model. Factors with significance were included in the multivariate analysis. All statistical analyses were performed using open-source R 4.1.2 (The R Foundation for Statistical Computing, Vienna, Austria).

## 3. Results

### 3.1. Patients

In total, 35 patients with eight primary lesions and 92 relapsed or metastatic lesions were included in this study. The median follow-up time was 17 months (range 6.1–75.4 months). The patient baseline characteristics are listed in Table 1. Specifically, 29 cases (82.9%) were clear cell carcinoma, 88.5% of the patients had an intermediate or poor International Metastatic Renal Cell Carcinoma Database Consortium (IMDC) risk, and 60% were on second- to fourth-line systemic therapy.

Surgery on primary or metastatic sites was previously performed in most patients (85.7%). The median time interval from first distant metastasis to radiation therapy was 7.2 months (range from 0 to 50.2 months). Of the patients, 19 were treated for all primary and/or metastatic lesions. Reasons for why some patients did not receive irradiation at all sites were discretions by treating physicians regarding radiation or immunotherapy-related AEs such as pneumonitis or the uncertainty of malignancy on diagnosis, among others. In total, 50 lesions were irradiated: 10 primary renal cancer, 14 bone, 2 adrenal, 7 lung, 8 lymph node, 4 liver, and 5 soft tissue. The range and the median value of the total volume irradiated was 29.9 (0.9–966.1) cc. Of the 50 irradiated lesions, all received high-dose SABR or SABR followed by conventional fractionated therapy. Forty percent of the patients received RT before tyrosine kinase inhibitor (TKI) failure. Table 2 summarizes the treatment characteristics correlated with RT.

### 3.2. Treatment Results

**Figure 1:**
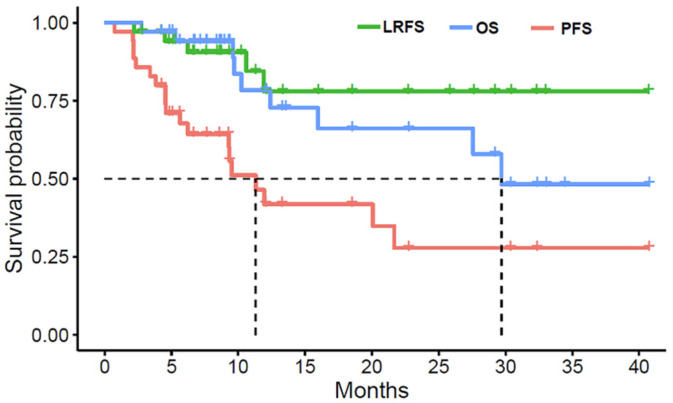
Local relapse-free survival (LRFS), progression-free survival rate (PFS), and overall survival rate (OS) for all patients.

The LRFS rates at one and three years were both 78%. The median time to local relapse was not reached. The median PFS time was 11.3 months, and the one- and three-year PFS rates were 41.8% and 27.9%, respectively. The OS rates at one and three years were 78.3% and 48.2%, respectively. The median OS time was 29.7 months (Figure 1).

**Figure 2:**
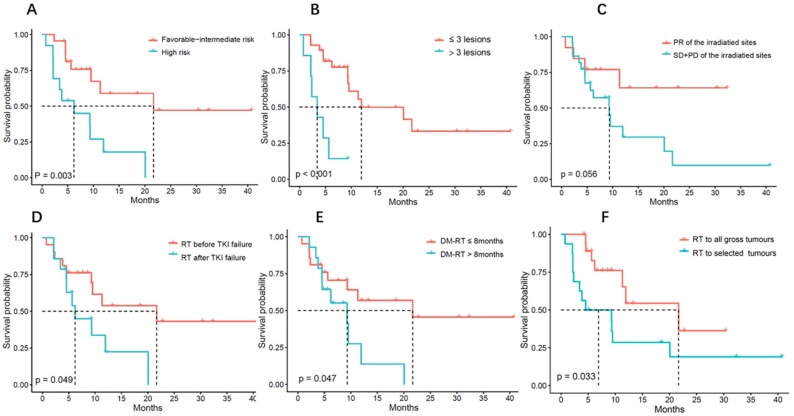
Progression-free survival rate (PFS) of oligometastatic renal cell carcinoma patients (*n* = 35) treated with SABR/P-SABR in relation to (**A**) IMDC risk, (**B**) number of metastases, (**C**) response to RT, (**D**) sequencing with TKI, (**E**) time from first metastasis to RT, and (**F**) radiation coverage.

**Figure 3:**
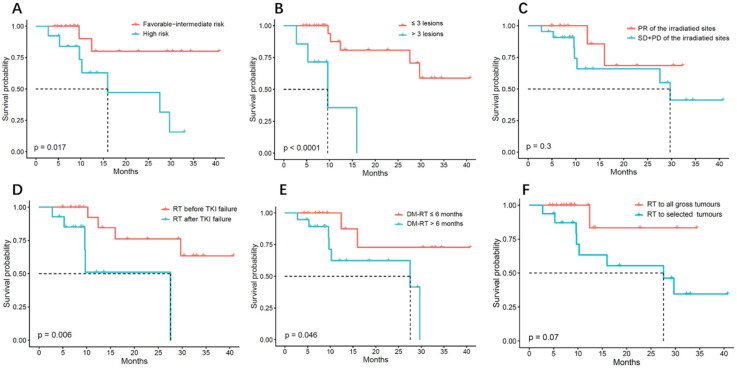
Overall survival rate (OS) of oligometastatic renal cell carcinoma patients (*n* = 35) treated with SABR/P-SABR in relation to (**A**) IMDC risk, (**B**) number of metastases, (**C**) response to RT, (**D**) sequencing with TKI, (**E**) time from first metastasis to RT, and (**F**) radiation coverage.

Regarding patient characteristics, favorable intermediate-risk IMDC scores (*p* = 0.003 and 0.017 for PFS and OS, respectively) and fewer (≤3) metastatic lesions (*p* < 0.001 for both PFS and OS) were significantly associated with better PFS and OS (Figure 2A,B and Figure 3A,B). It seems that patients with a good response to RT also showed improvement in LRFS (*p* = 0.054) (Supplementary Figure S1C). Interestingly, no progression was found for irradiated lesions in six metastatic non-clear cell renal cell carcinoma (nccRCC) patients.

Regarding the treatment characteristics, radiation before TKI failure and a short time interval from DM-RT were significant prognostic factors for PFS (Figure 2D,E). The median PFS was 21.7 months for patients who received RT before TKI failure and 6.2 months for those who did not (*p* = 0.049). The median PFS was 21.7 months and 9.3 months for patients who underwent RT within eight months and for those with a longer time interval (*p* = 0.047), respectively. Similarly, radiation before TKI failure (*p* = 0.006) and a short time interval (<6 months) from DM-RT (*p* = 0.046) were also associated with a significant improvement in OS (Figure 3D,E).

An additional significant predictor of improved PFS was radiation coverage. The median PFS was 21.7 months in patients whose tumors were all irradiated and 4.5 months for patients who received irradiation for selected tumors (*p* = 0.033) (Figure 2F). There was also a trend showing that the irradiation of all tumor sites was a favorable factor for OS (*p* = 0.07) (Figure 3F).

In multivariate analysis, an increasing number of metastases was associated with shorter PFS and OS (*p* = 0.001 and 0.003, respectively). SABR before TKI failure was also a good prognostic factor for OS (*p* = 0.028). However, no significant difference was found for other characteristics due to the small sample size. Supplementary Table S1 lists the HRs of PFS and OS for all characteristics.

### 3.3. Toxicity Profile

Among 35 patients, the most frequent grade 1–2 toxicities were gastrointestinal side effects in the irradiated area (15 patients) and anemia (eight patients). Only one case of grade 3 anemia was reported. No grade IV–V toxicities occurred (Table 3).

## 4. Discussion

The present study of our oligometastatic RCC cases in the era of targeted agents shows an 11.3-month PFS after SABR, in which 89% had IMDC intermediate- to high-risk diseases. The early use of SABR could result in a better prognosis. Moreover, we introduced a novel treatment regimen, P-SABR, for bulky lesions close to critical organs.

Several randomized studies have recently confirmed the existence of the oligometastatic state and demonstrated the efficacy of SABR for improving survival and extending ongoing systemic therapy [12,13]. A recent systematic review provided evidence that SABR in combination with targeted therapies or immune checkpoint inhibitors might be a very attractive therapeutic strategy in mRCC (due to its peculiar microenvironment and immunogenic features), showing promising clinical outcomes [14]. Emerging in vitro evidence indicates that high-dose radiation could efficiently eradicate RCC cells [15]. High-dose single-fraction RT can overcome the radioresistance of RCC, and SABR, which delivers high-dose fraction irradiation, has shown high LRFS rates for both primary [16] and metastatic RCC [17]. One study showed that the control rates among patients treated with a dose of 24 Gy in a single fraction were superior to the hypofractionation group (3–5 fraction regimen). However, in this study, the mean PTV volume in the single-fraction group was half that in the hypofractionation group (121.4 vs. 232.1 cm^3^, *p* = 0.03) [18]. For tumors of a large volume or adjacent to critical organs, it is sometimes unrealistic to deliver a single high dose or even hypofractionated radiation. Thus, treatment regimens should be personalized, and a regimen balancing the benefits and risks of high-dose radiation (i.e., P-SABR) may have value. The advantages of P-SABR are multiple. First, it not only protects the OAR from high-dose radiation but also delivers a high BED to a maximum target volume in the tumor center. Second, the periphery of the tumor itself could be a natural spacer during the first 3–5 fractions with SABR. Satisfactory outcomes after P-SABR for large lung cancers have been reported [8].

Regarding the dose and irradiation sites, all our patients received multiple- or all-site irradiation with high-dose SABR or P-SABR. We achieved a PFS time of 11.3 months even in this group of patients in which most had intermediate- or poor-risk disease and were on second- to fourth-line therapy. The NIVES [19] and RADVAX [20] studies investigated the efficacy of combining SABR with immunotherapy in mRCC. One or two of the metastatic lesions received a dose of 10 Gy over three or five fractions. The disease control rate was high in both cases and the PFS was 5.6 and 8.2 months, respectively. We may consider that the comprehensive high-dose irradiation of multiple or all lesions has brought forward the likelihood of obtaining high local and disease control and maximizing the effect on the stimulation of the immune system [21]. The RAPPORT trial evaluated the outcomes of total metastatic irradiation with immunotherapy and resulted in a relatively long PFS of 15.6 months [22]. Our study also showed that patients with all tumor sites covered by radiotherapy had significantly higher PFS rates than those where they were not. Meanwhile, the addition of SABR to systemic therapy did not lead to higher toxicities or therapy interruption. The SABR-COMET study showed that SABR to all metastases could improve survival [13], and a randomized phase-III trial for the treatment of 4–10 metastatic sites is currently being conducted [23].

For the timing of radiotherapy, a recent retrospective study by He et al. showed that the complete response rate was significantly higher before TKI failure in a group of patients with mRCC who were treated with SABR combined with targeted therapy [24]. Another study showed that the control of treated metastases positively correlated with the use of SABR before systemic therapies [25]. Our study also demonstrated that RT before the failure of TKI may achieve better survival outcomes. Stenman et al. investigated the effects of SABR or surgical metastasectomy in patients with mRCC and found that a watchful waiting period of 18 months or longer for local therapy was associated with better OS [26], which is inconsistent with our data, in which the time from DM to RT initiated before six months was found to be superior in terms of OS. Emphasis should be put on the fact that in retrospective studies, especially when RT is not routinely recommended as an upfront local treatment for mRCC patients, RT may apply to patients with a higher risk of local relapse or further distant metastasis. Patients with a short interval from diagnosis of DM to RT may have tumors with more aggressive characteristics [27]. In addition, in this study, OS was better when the intent was curative and one of the definitions of curative treatment was upfront local treatment [26]. Another study of front-line SABR with curative intent for mRCC patients also showed excellent results, with two-year LRFS and OS rates of 91.5% and 84.8%, respectively. In a further study, the median time to SBRT was 55 months [15], with PFS at 12 months of only 46.2%. Finally, some patients might not tolerate TKIs, therefore, the SABR of metastasis is important before the discontinuation or dose reduction in TKIs that might lead to further tumor progression or spreading.

It is interesting to note that in our study, all nccRCC lesions had good responses and achieved great local control after RT. Most of them were papillary renal cell cancers. A case report showed that a patient with papillary renal cell cancer achieved a complete response (CR) after only 27 Gy in three fractions [28]. Another study showed no microscopic residual disease after the preoperative irradiation of 16 Gy in four fractions for a papillary-type case [29]. The radiosensitivity of RCC cells with different pathologies may be a potential direction for future studies to investigate.

As previously reported in retrospective trials, patients with one to three metastases treated with SABR had better local control and a longer survival time than patients with more than three metastases [25,30]. Additionally, patients with intermediate- or high-risk disease have been proven to have worse outcomes than those with low-risk disease [11,12], which is consistent with our data.

There are several limitations to this study. The retrospective design inevitably introduced bias in the selection of treatment regimens. However, we still believe that this study reflects a real-world situation. High-quality evidence from prospective studies is needed.

## 5. Conclusions

We evaluated the long-term survival and toxicities after high-dose SABR and systemic therapy in oligometastatic RCC patients. The early use of SABR with high-dose irradiation may be safe and effective. P-SABR is a reasonable and flexible regimen for treating bulky lesions close to critical organs.

## Figures and Tables

**Table 1:** Patient baseline characteristics.

Characteristic	Value
Age (y), median (range)	63 (32–82)
Sex	
Male	29 (82.9)
Female	6 (17.1)
ECOG ^a^ score	
0	4 (11.4)
1	31 (88.6)
Number of lesions	
≤3	28 (80.0)
>3	7 (20.0)
Pathological type	
Clear-cell carcinoma	29 (82.9)
Papillary renal carcinomas	4 (17.1)
Others	2 (5.7)
Sarcomatoid differentiation	4 (11.4)
IMDC ^b^ score	
Favorable risk	4 (17.1)
Intermediate risk	18 (51.4)
High risk	13 (37.1)
Synchronous metastases	9 (25.7)
Surgery	
No surgery	5 (14.3)
RN ^c^ or NSS ^d^ only	22 (62.9)
Metastasectomy only	1 (2.9)
Metastasectomy plus RN or NSS	7 (20.0)
Tyrosine kinase inhibitor	31 (88.6)
PD-1 ^e^ inhibitor	10 (28.6)

Abbreviations: ^a^ ECOG, Eastern Cooperative Oncology Group; ^b^ IMDC, International Metastatic Renal Cell Carcinoma Database Consortium; ^c^ RN, radical nephrectomy; ^d^ NSS, nephron-sparing surgery for renal cancer; ^e^ PD-1, programmed death-1.

**Table 2:** Treatment characteristics that correlated with radiation therapy.

Treatment Characteristics	
Time interval from first distant metastasis to radiation therapy	
Within 6 months	16 (45.7)
Within 8 months	21 (60.0)
Sequencing with TKI ^a^	
Before failure of TKI	14 (40.0)
After failure of TKI	21 (60.0)
Coverage of irradiated sites	
Selected tumor sites irradiation	16 (45.7)
All tumor sites irradiation	19 (54.3)
Response of the irradiation sites	
PR ^b^	13 (37.1)
SD ^c^ or PD ^d^	22 (62.9)

Abbreviations: ^a^ TKI, tyrosine kinase inhibitor; ^b^ PR, partial response; ^c^ SD, stable disease; ^d^ PD, progressive disease.

**Table 3:** Toxicities in mRCC patients from stereotactic radiotherapy.

Side Effects	Grade 1	Grade 2	Grade 3
Anemia	5	3	1
Pneumonitis	1	2	-
Gastrointestinal toxicity	12	3	-
Dermatitis	3	-	-
Neutropenia	1	-	-
Thrombocytopenia	1	-	-
Fistula	-	1	-

## Data Availability

The datasets generated during and/or analyzed during the current study are available from the corresponding author upon reasonable request.

## Supplementary Materials

The following supporting information can be downloaded at https://www.mdpi.com/article/10.3390/curroncol29100619/s1. Supplementary Figure S1: Local relapse-free survival (LRFS) of oligometastatic renal cell carcinoma patients (*n* = 35) treated with SABR/P-SABR in relation to (A) IMDC risk, (B) number of metastases, (C) response to RT, (D) sequencing with TKI, (E) time from first metastasis to RT, and (F) radiation coverage. Supplementary Table S1: Univariate and multivariate analysis for PFS and OS.
