# Supplementary material for: Outcomes of High-Dose Stereotactic Ablative Radiotherapy to All/Multiple Sites for Oligometastatic Renal Cell Cancer Patients
Source: Curr Oncol. 2022 Oct 17;29(10):7832–41. doi: 10.3390/curroncol29100619 (PMC9600736; doi:10.3390/curroncol29100619)
Supplement: Supplementary file 1 [file curroncol-29-00619-s001.zip › curroncol-1880443-supplementary.pdf]

Current Oncology

Article

Outcomes of High-Dose Stereotactic Ablative Radiotherapy to All/Multiple Sites for Oligometastatic Renal Cell Cancer Patients

Ming-Wei Ma, Hong-Zhen Li, Xian-Shu Gao, Ming-Zhu Liu, Huan Yin, Kai-Wei Yang, Jia-Yan Chen, Xue-Ying Ren and Dian Wang

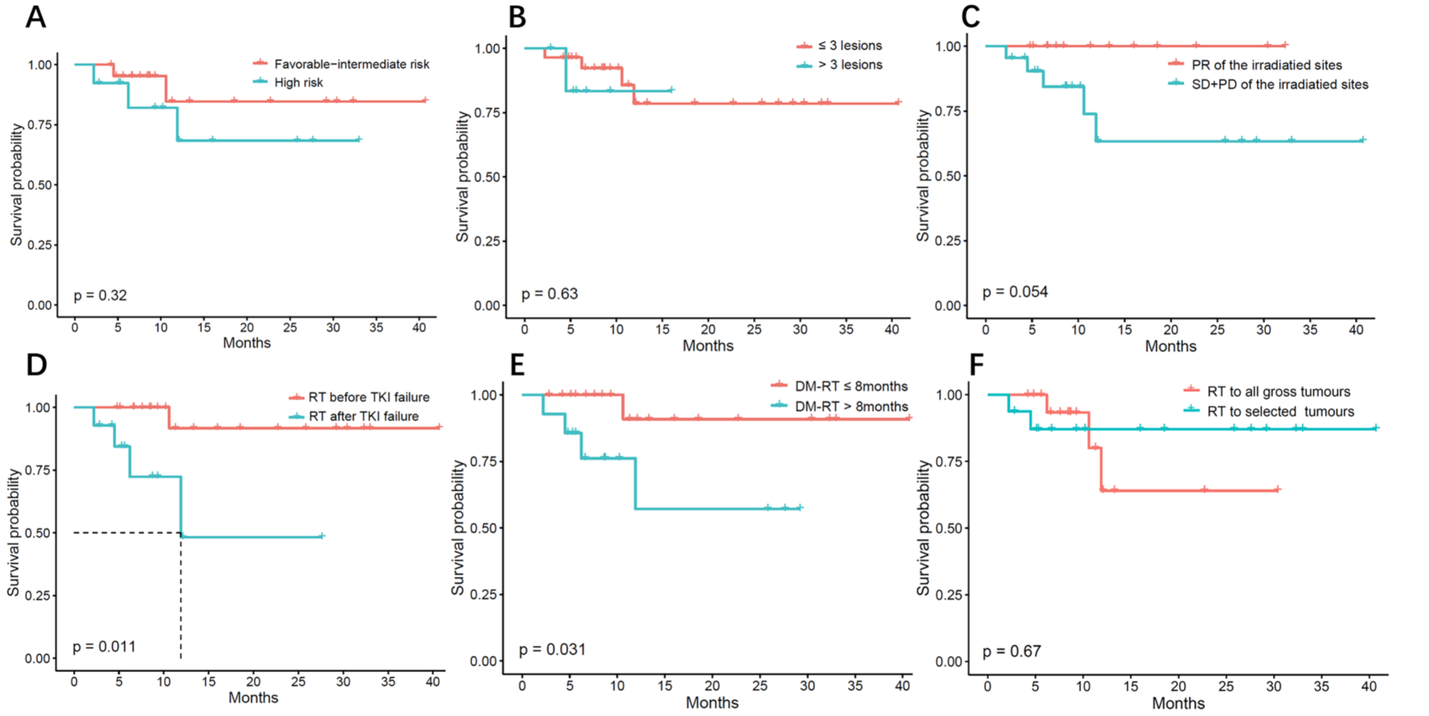

Supplementary Figure S1. Local relapse-free survival (LRFS) of oligometastatic renal cell carcinoma patients (n = 35) treated with SABR/P-SABR in relation to (**A**) IMDC risk, (**B**) number of metastases, (**C**) response to RT, (**D**) sequencing with TKI, (**E**) time from first metastasis to RT, and (**F**) radiation coverage.

Supplementary Table S1. Univariate and multivariate analysis for PFS and OS.

	**Univariate analysis for PFS** [a]		**Multivariate analysis for PFS**		Univariate analysis for OS [b]		Multivariate analysis for OS	
	**HR** [c] **(95% CI)**	**P value**	**HR (95% CI)**	P value	HR (95% CI)	P value	HR (95% CI)	P value
**Gender**								
Male	1 (Ref)				1 (Ref)			
Female	1.16 (0.38-3.52)	0.800			1.71 (0.42-6.88)	0.452		
**Age**								
<65	1 (Ref)				1 (Ref)			
≥65	0.40 (0.13-1.19)	0.097			0.755 (0.19-3.05)	0.693		
**IMDC** [d] **score**								

Favorable - intermediate risk	1 (Ref)				1 (Ref)			
High risk	3.88 (1.49-10.07)	**0.005**	4.46 (1.54-12.94)	**0.006**	5.56 (1.15-26.86)	**0.033**	2.82 (0.51-15.69)	0.235
**Pathology**								
Clear cell carcinoma	1 (Ref)				1 (Ref)			
Other pathological type	0.42 (0.10-1.85)	0.253			0.04 (0.00-56.63)	0.373		
**Presence of metastases**								
Metachronous metastases	1 (Ref)				1 (Ref)			
Synchronous metastases	1.11 (0.40-3.08)	0.847			1.39 (0.35-5.63)	0.642		
**Number of metastatic lesions**								
≤3	1 (Ref)				1 (Ref)			
4-5	5.70 (1.92-16.86)	**0.002**	8.97 (2.58-31.18)	**0.001**	12.07 (2.57-56.64)	**0.002**	17.06 (2.66-109.55)	**0.003**
**Sequencing with TKI** [e]								
Before failure of TKI	1 (Ref)				1 (Ref)			
After failure of TKI	2.49 (0.97-6.40)	0.057			6.53 (1.46-29.18)	**0.014**	7.80 (1.24-49.06)	**0.028**
**Time interval from first distant metastasis to radiation therapy**								
> the following months	1 (Ref)				1 (Ref)			
> 6 months	1.68 (0.65-4.34)	0.285			4.64 (0.91-23.72)	0.065		
> 8 months	2.54 (0.98-6.61)	0.055			2.76 (0.73-10.51)	0.136		
>12 months	2.60 (0.99-6.84)	0.053			3.28 (0.86-12.49)	0.082		
**Use of PD-1** [f] **inhibitor**								
No	1 (Ref)							
Yes	1.15 (0.44-3.04)	0.778			1.02 (0.21-4.97)	0.981		
**Response of the irradiation sites**								
PR [g]	1 (Ref)				1 (Ref)			
SD [h] + PD [i]	2.81 (0.93-8.54)	0.068			2.26 (0.47-11.01)	0.312		
**Coverage of irradiation sites**								
Full coverage	1 (Ref)				1 (Ref)			
Partial coverage	2.66 (1.04-6.80)	**0.041**	0.55 (0.20-1.53)	0.253	5.65 (0.70-45.95)	0.106		

Abbreviations: [a] PFS, progression-free survival; [b] HR, Hazard ratios; [c] OS, overall survival; [d] IMDC, International Metastatic Renal Cell Carcinoma Database Consortium; [e] TKI, tyrosine kinase inhibitor; [f] PD-1, programmed death-1; [g] PR, partial response; [h] SD, stable disease; [i] PD, progressive disease.
